# Quality and Reporting of Diagnostic Accuracy Studies in TB, HIV and Malaria: Evaluation Using QUADAS and STARD Standards

**DOI:** 10.1371/journal.pone.0007753

**Published:** 2009-11-13

**Authors:** Patricia Scolari Fontela, Nitika Pant Pai, Ian Schiller, Nandini Dendukuri, Andrew Ramsay, Madhukar Pai

**Affiliations:** 1 Department of Epidemiology, Biostatistics and Occupational Health, McGill University, Montreal, Canada; 2 Department of Medicine, Division of Clinical Epidemiology, McGill University, Montreal, Canada; 3 Special Programme for Research and Training in Tropical Diseases, World Health Organization, Geneva, Switzerland; 4 Respiratory Epidemiology and Clinical Research Unit, Montreal Chest Institute, Montreal, Canada; University of Stellenbosch, South Africa

## Abstract

**Background:**

Poor methodological quality and reporting are known concerns with diagnostic accuracy studies. In 2003, the QUADAS tool and the STARD standards were published for evaluating the quality and improving the reporting of diagnostic studies, respectively. However, it is unclear whether these tools have been applied to diagnostic studies of infectious diseases. We performed a systematic review on the methodological and reporting quality of diagnostic studies in TB, malaria and HIV.

**Methods:**

We identified diagnostic accuracy studies of commercial tests for TB, malaria and HIV through a systematic search of the literature using PubMed and EMBASE (2004–2006). Original studies that reported sensitivity and specificity data were included. Two reviewers independently extracted data on study characteristics and diagnostic accuracy, and used QUADAS and STARD to evaluate the quality of methods and reporting, respectively.

**Findings:**

Ninety (38%) of 238 articles met inclusion criteria. All studies had design deficiencies. Study quality indicators that were met in less than 25% of the studies included adequate description of withdrawals (6%) and reference test execution (10%), absence of index test review bias (19%) and reference test review bias (24%), and report of uninterpretable results (22%). In terms of quality of reporting, 9 STARD indicators were reported in less than 25% of the studies: methods for calculation and estimates of reproducibility (0%), adverse effects of the diagnostic tests (1%), estimates of diagnostic accuracy between subgroups (10%), distribution of severity of disease/other diagnoses (11%), number of eligible patients who did not participate in the study (14%), blinding of the test readers (16%), and description of the team executing the test and management of indeterminate/outlier results (both 17%). The use of STARD was not explicitly mentioned in any study. Only 22% of 46 journals that published the studies included in this review required authors to use STARD.

**Conclusion:**

Recently published diagnostic accuracy studies on commercial tests for TB, malaria and HIV have moderate to low quality and are poorly reported. The more frequent use of tools such as QUADAS and STARD may be necessary to improve the methodological and reporting quality of future diagnostic accuracy studies in infectious diseases.

## Introduction

Tuberculosis (TB), malaria and human immunodeficiency virus (HIV), the ‘big three’ among infectious diseases, are major global causes of morbidity and mortality. Together, they cause more than 3.5 million deaths per year.[1], [2], [3] Consequently, considerable financial and other investments have been directed towards the control of these diseases in recent years, which includes the development of diagnostic and treatment services that are accessible to patients. For example, the Global Fund to Fight AIDS, TB and Malaria has committed US$ 15.6 billion in 140 countries to support large-scale prevention, treatment and care programs against these three diseases.[4]

Recently, simple and robust technological platforms that allow rapid diagnostic testing at the primary health care level have greatly increased diagnostic capability, particularly in developing countries. The use of such tests for HIV is well-established, and the use of rapid diagnostic tests (RDT) in malaria control programmes is increasing.[5], [6] Although point-of-care (POC) tests for TB have not been successful, the WHO has recently endorsed the use of two new diagnostic technologies for TB and drug-resistance, and several other new TB diagnostics are in the pipeline. [7], [8], [9], [10]

The increasing number of diagnostic tests for TB, malaria and HIV leaves regulatory authorities, policy makers and health care professionals with the difficult task of choosing the tests that would best fit their patient populations and health-care delivery systems. In order to make evidence-based decisions, they often use published diagnostic accuracy studies as a way of gathering evidence about their options. [8] Also, the Grading of Recommendations Assessment, Development and Evaluation (GRADE) approach to guideline development requires a careful assessment of evidence on diagnostic accuracy, as well as other considerations, such as patient-important outcomes, the overall quality of evidence across these outcomes and the balance between benefits and harms and the strength of recommendations. [11], [12] However, systematic reviews have revealed that the value of diagnostic accuracy studies is frequently compromised by poor methodological quality and/or poor reporting.[13], [14], [15] There is also a growing realization that design flaws can systematically bias estimates of diagnostic accuracy.[16], [17], [18] Furthermore, even diagnostic test accuracy data may not be sufficient for policy making, because they are surrogates for patient-important outcomes.[12]

In 2003, two tools were developed with the objective of providing researchers with a standardized and validated format for assessing quality of diagnostic studies and a template for improving reporting: QUADAS (Quality Assessment of Studies of Diagnostic Accuracy) and STARD (STAndards for the Reporting of Diagnostic accuracy studies).[19], [20], [21] QUADAS was designed to be used in systematic reviews to evaluate the quality of primary diagnostic accuracy studies, while STARD was developed to improve the quality of reporting of diagnostic accuracy studies in general.

Both tools are slowly gaining acceptance in the diagnostic literature. In April 2008, it was estimated that more than 200 biomedical journals encouraged the use of the STARD statement in their instructions for authors.[22] The QUADAS tool is increasingly being used in diagnostic accuracy meta-analyses. However, it is unclear if these tools have been widely accepted and applied to diagnostic accuracy studies of major infectious diseases. We performed a systematic review with the objective to describe the methodological and reporting quality of recently published diagnostic accuracy studies on commercial tests for TB, malaria and HIV.

## Methods

### Search Strategy

We searched PubMed and EMBASE (OVID interface) for primary diagnostic accuracy studies published between January 2004 and December 2006. We chose these databases because together they have a wide coverage of the health literature and would therefore enable us to obtain a fairly representative sample of indexed diagnostic studies published in the time period of interest. We limited the search to the period between 2004 and 2006 because we wanted to determine the methodological and reporting quality of diagnostic studies following the publication and dissemination of QUADAS and STARD.

The keywords and search terms that were used included {[‘tuberculosis’ (explode) OR ‘*Mycobacterium tuberculosis’* (explode) OR ‘(tuberculosis or tuberculous).ti’] OR [‘malaria’ (explode) OR ‘Plasmodium’ (explode) OR ‘malaria.ti’] OR [‘HIV’ (explode) OR ‘HIV seropositivity’ (explode) OR ‘HIV infections’ (explode) OR ‘acquired immunodeficiency syndrome’ (explode) OR ‘HIV.ti’]} AND [‘sensitivity and specificity’ (explode) OR ‘specificity.ti’ OR ‘specificity.ab’ OR ‘accuracy.ti’ OR ‘diagn$.ti’]}. The search was limited to studies in humans.

### Study Eligibility

We included diagnostic accuracy studies on commercial tests for TB, malaria and HIV that aimed to determine sensitivity and specificity of a given diagnostic test for one of these three infections. To be eligible, the studies had to be original, describe their methods, report sensitivity and specificity data and be published between January 2004 and December 2006. Languages were restricted to English, French, Spanish and Portuguese (languages that our study team was able to cover). Because commercial tests are standardized and usually test methods are well reported and easily defined, we restricted the study to commercial kits. In addition, commercial tests are more likely to be used in routine clinical practice than exclusively for research.

### Study Selection

Initially, one reviewer (PSF) screened the titles and abstracts of the citations retrieved by the electronic search (first screen). Citations that were identified as diagnostic accuracy studies were classified according to the disease (TB, malaria or HIV).

**Figure 1:**
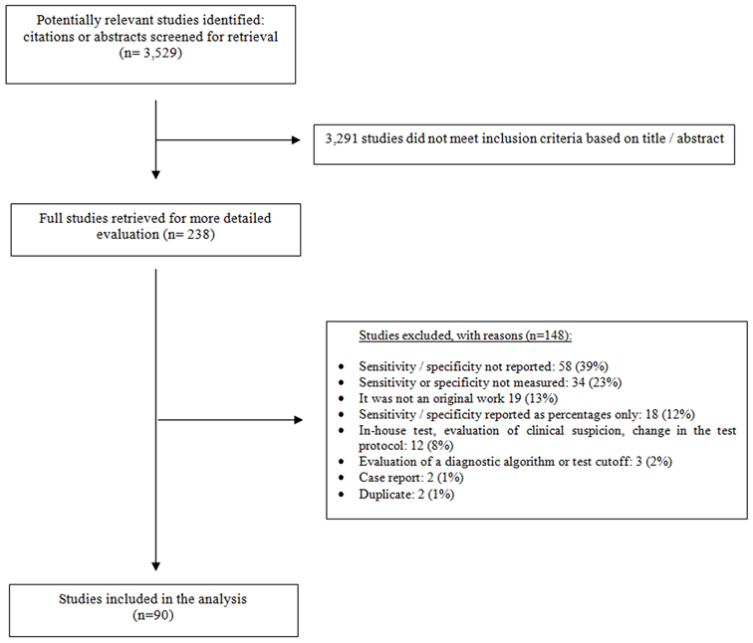
Flow diagram for study selection.

One researcher (PSF) reviewed the full text of all potentially eligible studies. A second researcher (NPP) independently reviewed 20% of all full text articles considered relevant in the first screen. Disagreements among reviewers were resolved by consensus. Figure 1 describes the study selection process.

### Data Abstraction

Two researchers (MP and PSF) created a data extraction form to be used in this review. The initial form was piloted by two reviewers (PSF and NPP) with 5% of the included publications. Based upon experience gained in the pilot, we modified and finalized the data extraction form.

Data extracted only included information explicitly stated in the text. Data retrieved included the following: year of publication, journal, disease of interest, type of commercial diagnostic test, reference standard employed, and data on quality of methods and reporting (listed below). When data were unavailable or not stated explicitly, the reviewers coded the information as “not reported”. Any remaining disagreements were resolved by consensus before finalizing the data extraction.

### Assessment of Methodological Quality

We assessed the methodological quality of studies using QUADAS.[20], [21] QUADAS is a validated quality checklist composed of 14 items, which encompass the most important sources of bias and variation observed in diagnostic accuracy studies. It was developed using a Delphi procedure which was used to reduce an initial list of 28 quality items.

The quality assessment items included in QUADAS are: spectrum composition, description of selection criteria and reference standard, disease progression bias, partial and differential verification, incorporation bias, description of index and reference test execution, test and reference standard review bias, clinical review bias, and description of uninterpretable test results. The definition of the items listed above can be found in Table 1. All the researchers involved in data extraction (PSF and NPP) were trained in the use of QUADAS checklist. Each item in the QUADAS checklist was scored as “Yes”, “No”, or “Unclear”, as per the recommendations of the authors of the QUADAS checklist.

**Table 1:** Biases in diagnostic accuracy test studies.

Bias	Definition
Spectrum composition bias	When the spectrum of patients is not representative of the patients who will receive the test in practice
Disease progression bias	When the time period between reference standard and index test is not short enough to be reasonably sure that the target condition did not change between the two tests
Partial verification bias	When the whole sample or a random selection of the sample does not receive verification using a reference standard of diagnosis
Differential verification bias	When patients receive different reference standard depending on the index test result
Incorporation bias	When the reference standard is not independent of the index test, i.e., when the index test forms part of the reference standard
Test review bias	When the index test results are interpreted with knowledge of the results of the reference standard
Reference standard review bias	When the reference standard test results are interpreted with knowledge of the results of the index test
Clinical review bias	When test results are interpreted in the light of the clinical data that would not be available when the test is used in practice

Adapted from: Whiting P, Rutjes A, Reitsma J, Bossuyt P, Kleijnen J. The development of QUADAS: a tool for the quality assessment of studies of diagnostic accuracy included in systematic reviews. BMC Medical Research Methodology 2003;3:25.

### Assessment of Quality of Reporting

The quality of the reporting was evaluated using the STARD criteria.[19] STARD, developed by a group of scientists and editors, consists of a checklist of 25 items that assess the completeness of reporting in diagnostic studies, potential sources of bias and generalizability. The checklist is subdivided in 5 sections: title/abstract/keywords, introduction, methods, results, and discussion. The majority of items in the STARD checklist were scored as “Not reported” or “Reported”. The “Reported” category included both “Fully reported” and “Partially reported” sub-categories. A “Partially reported” item means that the authors mentioned the item, but did not provide all the information required by the STARD checklist about it.

Three STARD items were scored using other criteria: the item “participant recruitment” was scored as “recruitment based on symptoms” or “other recruitment/unclear”, while the item “participant sampling” was classified as “consecutive sampling” or “other sampling strategy/unclear”. Finally, the item “data collection” was scored as “prospective” and “retrospective”.

Eight out of the 25 STARD reporting items were considered essential by our group for the purposes of our project: reporting of the sampling strategy used, reference standard test, data collection methods, blinding, proportion of eligible patients that did not participate in the study, inclusion and exclusion criteria, participant recruitment and description of clinic and demographic characteristics of the study population. These items were used to compare the quality of reporting of studies after stratifying them by disease (TB, Malaria and HIV).

### Use of STARD

In order to determine the frequency of use of STARD in diagnostic accuracy studies, we searched the full-text of all the included papers for any explicit mention of their use by the authors. Furthermore, in September 2008, we accessed the sections containing “information for the authors” (author guidelines) on the websites of all the journals (46 in all) in which the included papers were published. In doing so, we wanted to determine if the use of STARD was required when submitting a diagnostic accuracy manuscript to these journals.

### Data Synthesis and Statistical Analysis

Descriptive statistics were used to summarize the number and proportion of included studies that met the QUADAS and STARD criteria. We carried out a qualitative synthesis of the study characteristics, and quality of the methodology and reporting. Since the studies were heterogeneous with respect to diseases (TB, malaria and HIV), we decided to present overall results, as well as results stratified by disease subgroup. We also stratified the results by year of study publication in order to capture any temporal change since the publication of the STARD and QUADAS guidelines.

## Results

### Study Selection

We identified a total of 3,529 potentially relevant citations from the database searches. After the first and second screens, a total of 90 full-text studies were eligible for inclusion in this systematic review (Figure 1).

### Description of Included Studies

The characteristics of the included studies are shown in Table 2. Most papers were published in 2004 (47%). The 90 studies included were published in 46 different medical journals, Fifty percent evaluated TB diagnostic tests, 21% malaria diagnostic tests, and 29% HIV diagnostic tests.

**Table 2:** Characteristics of the studies included (N = 90).

Characteristic	Frequency (%)
**Disease**	
Tuberculosis	45 (50)
Malaria	18 (20)
HIV	27 (30)
**Studies' origin** *	
Africa	16
Asia	29
Australia and Oceania	01
Europe	27
North America	11
South America	06
**Number of patients per study**	
Median (interquartile range)	209 (110–555)
**Number of studies with industry involvement**	39 (43)
**Number of studies with conflict of interest**	38 (42)
**Year of publication**	
2004	42 (47)
2005	21 (23)
2006	27 (30)
**Number of journals where included studies were published**	46

* The total number of countries is not 90 because there were some studies that were performed in more than one country.

### Use of STARD

No study explicitly mentioned using STARD for preparing the manuscript (this, however, does not mean that this tool was not actually used). When the journal websites of the 46 journals that published the included papers were searched in September 2008, only 10 of them (22%) required the authors to use STARD when submitting diagnostic accuracy study manuscripts.

### Assessment of the Methodological Quality Using QUADAS

The overall results of the quality assessment using QUADAS, as well as the results after stratification by disease and year of publication are presented in Tables 3 and 4.

**Table 3:** Assessment of methodological quality using QUADAS* stratified by disease.

QUADAS item(scored as “Yes”)	Disease			Total
	Tuberculosis (N = 45)	Malaria (N = 18)	HIV (N = 27)	(N = 90)
	n (%)	n (%)	n (%)	n (%)
**QUADAS 1**				
Adequate spectrum composition	26 (58)	13 (72)	17 (63)	56 (62)
**QUADAS 2**				
Clear description of selection criteria	21 (47)	12 (67)	13 (48)	46 (51)
**QUADAS 3**				
Adequate reference standard	44 (98)	18 (100)	24 (89)	86 (96)
**QUADAS 4**				
Absence of disease progression bias	42 (93)	15 (83)	21 (78)	78 (87)
**QUADAS 5**				
Absence of partial verification bias	44 (98)	17 (94)	22 (81)	83 (92)
**QUADAS 6**				
Absence of differential verification bias	42 (93)	17 (94)	17 (63)	76 (84)
**QUADAS 7**				
Absence of incorporation bias	45 (100)	18 (100)	25 (93)	88 (98)
**QUADAS 8**				
Adequate description of the index test execution	15 (33)	3 (17)	7 (26)	25 (28)
**QUADAS 9**				
Adequate description of the reference test execution	6 (13)	2 (11)	1 (4)	9 (10)
**QUADAS 10**				
Absence of index test review bias	6 (13)	5 (28)	6 (22)	17 (19)
**QUADAS 11**				
Absence of reference test review bias	7 (16)	8 (44)	7 (26)	22 (24)
**QUADAS 12**				
Absence of clinical review bias	14 (31)	12 (67)	8 (30)	34 (38)
**QUADAS 13**				
Report of uninterpretable results	9 (20)	1 (6)	10 (37)	20 (22)
**QUADAS 14**				
Description of withdrawals	3 (7)	1 (6)	1 (4)	5 (6)

* Whiting P, Rutjes A, Reitsma J, Bossuyt P, Kleijnen J. The development of QUADAS: a tool for the quality assessment of studies of diagnostic accuracy included in systematic reviews. BMC Medical Research Methodology 2003;3:25.

**Table 4:** Assessment of methodological quality using QUADAS* stratified by year of publication.

QUADAS item (scored as “Yes”)	Year			Total
	2004 (N = 42)	2005 (N = 21)	2006 (N = 27)	(N = 90)
	n (%)	n (%)	n (%)	n (%)
**QUADAS 1**				
Adequate spectrum composition	26 (62)	14 (67)	16 (59)	56 (62)
**QUADAS 2**				
Clear description of selection criteria	21 (50)	12 (57)	13 (48)	46 (51)
**QUADAS 3**				
Adequate reference standard	41 (98)	20 (95)	25 (93)	86 (96)
**QUADAS 4**				
Absence of disease progression bias	38 (91)	16 (76)	24 (89)	78 (87)
**QUADAS 5**				
Absence of partial verification	40 (95)	17 (81)	26 (96)	83 (92)
**QUADAS 6**				
Absence of differential verification bias	36 (86)	16 (76)	24 (89)	76 (84)
**QUADAS 7**				
Absence of incorporation bias	42 (100)	19 (91)	27 (100)	88 (98)
**QUADAS 8**				
Adequate description of the index test execution	11 (26)	4 (19)	10 (37)	25 (28)
**QUADAS 9**				
Adequate description of the reference test execution	3 (7)	0 (0)	6 (22)	9 (10)
**QUADAS 10**				
Absence of index test review bias	10 (24)	4 (19)	3 (11)	17 (19)
**QUADAS 11**				
Absence of reference test review bias	10 (24)	3 (14)	9 (33)	22 (24)
**QUADAS 12**				
Absence of clinical review bias	17 (41)	7 (33)	10 (37)	34 (38)
**QUADAS 13**				
Report of uninterpretable results	10 (24)	4 (19)	6 (22)	20 (22)
**QUADAS 14**				
Description of withdrawals	2 (5)	0 (0)	3 (11)	5 (6)

* Whiting P, Rutjes A, Reitsma J, Bossuyt P, Kleijnen J. The development of QUADAS: a tool for the quality assessment of studies of diagnostic accuracy included in systematic reviews. BMC Medical Research Methodology 2003;3:25.

The majority of studies used an adequate reference standard test (96%), and did not suffer from incorporation and partial or differential verification biases (98 and 92%, respectively). Reference standard tests considered “adequate” for TB, malaria and HIV were, respectively, sputum culture, blood smear examination and ELISA and/or Western Blot. Nevertheless, all 90 studies included in this systematic review had at least one design flaw. The most commonly noted problems were associated with poor description of test execution, withdrawal of patients, and interpretation and reporting of test results.

Quality items that were reported in less than 25% of the studies included description of withdrawals (6%), adequate description of the reference test execution (10%), absence of index test review bias (19%), report of uninterpretable results (22%), and absence of reference test review bias (24%). Two other quality items were clearly described in less than 50% of the papers: index test execution (28%) and absence of clinical review bias (38%). Finally, a clear description of selection criteria and adequacy of spectrum composition, which are essential quality items for diagnostic accuracy studies, were reported in only 51 and 62% of studies, respectively.

Specific problems with some quality items were detected after we stratified the studies by disease (TB, malaria and HIV) and year of publication. In TB and HIV diagnostic accuracy studies, a clear description of selection criteria was present in less than 50% of time (47 and 48%, respectively). Moreover, the same item was reported in only 48% of the study sample published in 2006.

Furthermore, the results stratified by disease showed that HIV diagnostic accuracy studies met fewer of the methodological quality criteria when compared to those of TB and malaria. HIV studies were affected by higher prevalence of important biases such as partial (19%) and differential (37%) verification, incorporation (7%) and clinical review (70%) biases.

Finally, when the results were analyzed according to year of publication, we observed that in 2006, compared to previous years, a greater number of studies adequately described the index (37%) and reference standard (22%) tests used, as well as withdrawals (11%). These numbers, however, can still be considered very low.

### Assessment of the Quality of Report Using STARD

Tables 5 and 6 present the overall and stratified results (by disease and year of publication) in detail. No study fulfilled all the 25 items of STARD checklist. Overall, the major reporting problems encountered were in the sections about description of participants, test and statistical methods, and reporting of results.

**Table 5:** Assessment of the quality of report using STARD* stratified by disease.

Section and Topic in the STARD checklist (scored as “Reported”)	Disease			Total
	TB (N = 45)	Malaria (N = 18)	HIV (N = 27)	(N = 90)
	n (%)	n (%)	n (%)	n (%)
**TITLE/ABSTRACT/KEYWORDS**				
Identify the article as a study of diagnostic accuracy (recommend MeSH heading ‘sensitivity and specificity’).	44 (98)	18 (100)	27 (100)	89 (99)
**INTRODUCTION**				
State the research questions or study aims, such as estimating diagnostic accuracy or comparing accuracy between tests or across participant groups.	44 (98)	17 (94)	25 (93)	86 (96)
**METHODS** (describe)				
*Participants*				
The study population: the inclusion and exclusion criteria, setting and locations where the data were collected.	30 (67)	17 (94)	23 (85)	70 (78)
Participant recruitment: was recruitment based on presenting symptoms, results from previous tests, or the fact that the participants had received the index tests or the reference standard?^γ^	28 (62)	13 (29)	13 (48)	54 (60)
Participant sampling: was the study population a consecutive series of participants defined by the selection criteria in the previous 2 items? If not, specify how participants were further selected.^§^	14 (31)	8 (44)	6 (22)	28 (31)
Data collection: was data collection planned before the index test and reference standard were performed (prospective study) or after (retrospective study)?^θ^	38 (84)	16 (89)	21 (78)	75 (83)
*Test methods*				
The reference standard and its rationale.	45 (100)	18 (100)	25 (93)	88 (98)
Technical specifications of material and methods involved including how and when measurements were taken, and/or cite references for index tests and reference standard.	44 (98)	16 (89)	21 (78)	81 (90)
Definition of and rationale for the units, cutoffs, and/or categories of the results of the index tests and the reference standard.	41 (91)	16 (89)	18 (67)	75 (83)
The number, training, and expertise of the persons executing and reading the index tests and the reference standard.	3 (7)	7 (39)	5 (19)	15 (17)
Whether or not the readers of the index tests and reference standard were blind (masked) to the results of the other test and describe any other clinical information available to the readers.	5 (11)	6 (33)	3 (11)	14 (16)
*Statistical methods*				
Methods for calculating or comparing measures of diagnostic accuracy, and the statistical methods used to quantify uncertainty (e.g., 95% confidence intervals).	16 (36)	12 (67)	14 (52)	42 (47)
Methods for calculating test reproducibility, if done.	0 (0)	0 (0)	0 (0)	0 (0)
**RESULTS** (report)				
*Participants*				
When study was done, including beginning and ending dates of recruitment.	34 (76)	16 (89)	16 (59)	66 (73)
Clinical and demographic characteristics of the study population (e.g., age, sex, spectrum of presenting symptoms, comorbidity, current treatments, recruitment centers).	27 (60)	13 (29)	19 (70)	59 (66)
The number of participants satisfying the criteria for inclusion that did or did not undergo the index tests and/or the reference standard; describe why participants failed to receive either test (a flow diagram is strongly recommended).	7 (16)	4 (22)	2 (7)	13 (14)
*Test results*				
Time interval from the index tests to the reference standard, and any treatment administered between.	36 (80)	13 (29)	18 (67)	67 (74)
Distribution of severity of disease (define criteria) in those with the target condition; other diagnoses in participants without the target condition.	6 (13)	1 (6)	3 (11)	10 (11)
A cross tabulation of the results of the index tests (including indeterminate and missing results) by the results of the reference standard; for continuous results, the distribution of the test results by the results of the reference standard.	45 (100)	18 (100)	26 (96)	89 (99)
Any adverse events from performing the index tests or the reference standard.	1 (2)	0 (0)	0 (0)	1 (1)
*Estimates*				
Estimates of diagnostic accuracy and measures of statistical uncertainty (e.g., 95% confidence intervals).	43 (96)	17 (94)	27 (100)	87 (97)
How indeterminate results, missing responses, and outliers of the index tests were handled.	8 (18)	0 (0)	7 (26)	15 (17)
Estimates of variability of diagnostic accuracy between subgroups of participants, readers or centers, if done.	1 (2)	2 (11)	6 (22)	9 (10)
Estimates of test reproducibility, if done.	0 (0)	0 (0)	0 (0)	0 (0)
**DISCUSSION**				
Discuss the clinical applicability of the study findings.	44 (98)	18 (100)	27 (100)	89 (99)

TB  =  tuberculosis MeSH  =  medical subject heading ^γ^  =  recruitment based on symptoms ^§^  =  consecutive sampling ^θ^  =  prospective study.

* Adapted from Bossuyt PM, Reitsma JB, Bruns DE, et al. Towards complete and accurate reporting of studies of diagnostic accuracy: The STARD Initiative. Ann Intern Med 2003;138:40-4.

**Table 6:** Assessment of the quality of report using STARD* stratified by year of publication.

Section and Topic in the STARD checklist (scored as “Reported”)	Year			Total
	2004 (N = 42)	2005 (N = 21)	2006 (N = 27)	(N = 90)
	n (%)	n (%)	n (%)	n (%)
**TITLE/ABSTRACT/KEYWORDS**				
Identify the article as a study of diagnostic accuracy (recommend MeSH heading ‘sensitivity and specificity’).	42 (100)	21 (100)	26 (96)	89 (99)
**INTRODUCTION**				
State the research questions or study aims, such as estimating diagnostic accuracy or comparing accuracy between tests or across participant groups.	39 (93)	21 (100)	26 (96)	86 (96)
**METHODS** (describe)				
*Participants*				
The study population: the inclusion and exclusion criteria, setting and locations where the data were collected.	34 (81)	17 (81)	19 (70)	70 (78)
Participant recruitment: was recruitment based on presenting symptoms, results from previous tests, or the fact that the participants had received the index tests or the reference standard? ^γ^	24 (57)	13 (62)	17 (63)	54 (60)
Participant sampling: was the study population a consecutive series of participants defined by the selection criteria in the previous 2 items? If not, specify how participants were further selected.^§^	14 (33)	8 (38)	6 (22)	28 (31)
Data collection: was data collection planned before the index test and reference standard were performed (prospective study) or after (retrospective study)? ^θ^	36 (86)	18 (86)	21 (78)	75 (83)
*Test methods*				
The reference standard and its rationale.	45 (100)	18 (100)	25 (93)	88 (98)
Technical specifications of material and methods involved including how and when measurements were taken, and/or cite references for index tests and reference standard.	41 (98)	20 (95)	27 (100)	88 (98)
Definition of and rationale for the units, cutoffs, and/or categories of the results of the index tests and the reference standard.	38 (91)	19 (91)	24 (89)	81 (90)
The number, training, and expertise of the persons executing and reading the index tests and the reference standard.	34 (81)	17 (81)	24 (89)	75 (83)
Whether or not the readers of the index tests and reference standard were blind (masked) to the results of the other test and describe any other clinical information available to the readers.	8 (19)	3 (14)	4 (15)	15 (17)
*Statistical methods*				
Methods for calculating or comparing measures of diagnostic accuracy, and the statistical methods used to quantify uncertainty (e.g., 95% confidence intervals).	17 (41)	11 (52)	14 (52)	42 (47)
Methods for calculating test reproducibility, if done.	0 (0)	0 (0)	0 (0)	0 (0)
**RESULTS** (report)				
*Participants*				
When study was done, including beginning and ending dates of recruitment.	34 (81)	13 (62)	19 (70)	66 (73)
Clinical and demographic characteristics of the study population (e.g., age, sex, spectrum of presenting symptoms, comorbidity, current treatments, recruitment centers).	29 (69)	13 (62)	17 (70)	59 (66)
The number of participants satisfying the criteria for inclusion that did or did not undergo the index tests and/or the reference standard; describe why participants failed to receive either test (a flow diagram is strongly recommended).	7 (17)	2 (10)	4 (24)	13 (14)
*Test results*				
Time interval from the index tests to the reference standard, and any treatment administered between.	33 (79)	14 (67)	20 (74)	67 (74)
Distribution of severity of disease (define criteria) in those with the target condition; other diagnoses in participants without the target condition.	4 (10)	2 (10)	4 (24)	10 (11)
A cross tabulation of the results of the index tests (including indeterminate and missing results) by the results of the reference standard; for continuous results, the distribution of the test results by the results of the reference standard.	42 (100)	20 (95)	27 (100)	89 (99)
Any adverse events from performing the index tests or the reference standard.	1 (2)	0 (0)	0 (0)	1 (1)
*Estimates*				
Estimates of diagnostic accuracy and measures of statistical uncertainty (e.g., 95% confidence intervals).	40 (95)	20 (95)	27 (100)	87 (97)
How indeterminate results, missing responses, and outliers of the index tests were handled.	8 (18)	4 (19)	3 (11)	15 (17)
Estimates of variability of diagnostic accuracy between subgroups of participants, readers or centers, if done.	5 (12)	2 (10)	2 (7)	9 (10)
Estimates of test reproducibility, if done.	0 (0)	0 (0)	0 (0)	0 (0)
**DISCUSSION**				
Discuss the clinical applicability of the study findings.	41 (98)	21 (100)	27 (100)	89 (99)

MeSH  =  Medical Subject Heading ^γ^  =  recruitment based on symptoms ^§^  =  consecutive sampling ^θ^  =  prospective study.

* Adapted from Bossuyt PM, Reitsma JB, Bruns DE, et al. Towards complete and accurate reporting of studies of diagnostic accuracy: The STARD Initiative. Ann Intern Med 2003;138:40-4.

Nine STARD items were reported in less than 25% of the studies: methods for calculation and estimates of test reproducibility (0%), adverse effects of the diagnostic tests (1%), estimates of diagnostic accuracy between subgroups (10%), distribution of severity of disease/other diagnoses in study participants (11%), number of eligible patients who did not participate in the study (14%), blinding of the test readers (16%), and description of the team executing the test and management of indeterminate, invalid/outlier results (both 17%).

Two other STARD items were poorly reported (less than 50% of time): participant sampling method (31%) and statistical methods to calculate diagnostic accuracy and uncertainty/precision (47%). When specifically analyzing the reporting of results' uncertainty, we observed that only 22 of the studies (24%) presented 95% confidence intervals.

When stratifying the studies by disease, HIV diagnostic accuracy studies met fewer of the reporting standards compared to those of TB and malaria diagnostics. Reports of HIV diagnostic accuracy studies failed, more frequently, to describe 5 out of 8 reporting items considered essential by our group: sampling strategies used (reported in 22% of studies), reference standard test (reported in 93% of HIV studies compared to 100% in TB and malaria studies), data collection methods (reported in 78% of studies), blinding (reported in 11% of studies – same as malaria) and proportion of eligible patients that did not participate in the study (reported in only 7% of studies). The 3 other reporting items considered essential were inclusion and exclusion criteria, participant recruitment and description of clinic and demographic characteristics of the study population.

Analysis by year of publication, revealed that in 2006, a greater number of studies reported the recruitment strategies used (63%), technical specifications of material and methods (100%), characteristics of study population (70%), number of eligible patients that did not undergo index/reference standard test (24%), distribution of severity of disease (24%) and estimate of diagnostic accuracy and 95% confidence intervals (100%) compared to previous years. However, it is important to highlight that the more frequent reporting of items such as description of material and methods does not mean that the quality of the report was adequate.

## Discussion

TB, malaria and HIV are major killers with enormous global burden. High-quality evidence on diagnostics is critical for the development of evidence-based policies on diagnosis, and, ultimately, for effective control of these global epidemics.[23] In this study, we evaluated the methodological quality and reporting quality of recently published diagnostic accuracy studies in TB, HIV and malaria.

Our results show that diagnostic studies on TB, malaria and HIV commercial tests published between 2004 and 2006 had moderate to low methodological quality and were often poorly reported. Sources of bias and variation were present in all the studies, and important criteria for determining the presence of bias were often either not mentioned or unclearly reported. At least for TB and malaria, these results are consistent with previous observations made by several researchers.[8], [24], [25], [26]

Most worrisome is the fact that essential methodological elements, such as selection of a representative population and blinding, were not used and/or not reported by many researchers. Furthermore, only a small proportion of the studies adequately described the execution of both reference (10%) and index (28%) tests, and no study reported on reproducibility. The implications of the under-reporting of these elements are several. For example, the value of sensitivity and specificity estimates are unclear in the absence of clear information about test reproducibility. Moreover, if a reference standard is imperfect or poorly done, then this can potentially under-estimate or over-estimate the accuracy of a test. If the index test is poorly described, other researchers cannot replicate the study results (although this is less of an issue with standardized commercial kits).

### Strengths and Limitations

The major strength of this study is the systematic search for diagnostic accuracy studies via PubMed and EMBASE, two of the most widely used health literature databases. Furthermore, we used rigorous methods to select studies and abstract data, the latter independently conducted by two trained researchers.

The use of both QUADAS and STARD to evaluate diagnostic accuracy studies is also a strength of this systematic review. Both tools were developed by experts with the respective aims of assessing the quality of diagnostic studies included in systematic reviews and improve the quality of reporting of diagnostic studies in general. Furthermore, QUADAS and STARD are well standardized and easy to implement.[21], [27] The complementary aspect of these tools also allowed us to have a deeper understanding of the current methodological and reporting quality of these studies. For example, for the item “reference test execution”, while more than 90% of the studies reported the reference test execution (STARD), only less than 25% of them did it in an adequate and clear manner (QUADAS).

An important limitation of our study is that we did not compare our results to a sample of studies published before the publication of QUADAS and STARD instruments (i.e., prior to 2003). Consequently, we can provide information about the current quality of methods and reporting of diagnostic studies, but not about changes in quality or reporting over time.

Wilczynski and colleagues compared the quality of report of papers published in journals that endorsed STARD versus those that did not (i.e., journals that published or not the STARD statement in 2003).[28] Studies were also compared according to year of publication (2001, 2002, 2004 and 2005). The results showed that the quality of report was not affected by the type of journal, and that it remained similar over time.

Another limitation of our study is the fact that we decided to only record information that was clearly stated in the paper, coding as “not reported” when data were not available. Thus, it may be possible that methodological quality items were met in the actual study, but not reported. Because we did not contact all the authors, we were unable to resolve this issue.

### Implications

Poor quality of diagnostic studies is a recognized problem. After the publication of QUADAS and STARD in 2003, the expectation was that the methodological quality of diagnostic studies, and the quality of their reporting, would improve over the years. Unfortunately, this objective seems to be far from being achieved, at least with respect to diagnostic studies on major infectious diseases.

Our results suggest that STARD is probably not used by researchers as often as expected or desired, at least in the field of infectious diseases. Furthermore, we have shown that, based on the results of a search performed in September 2008, only 22% of the journals in our study sample required authors to use STARD when submitting a diagnostic accuracy manuscript for publication. Consequently, we hypothesize that fact that not many journals require authors to use STARD may be one of the causes behind the lack of improvement of reporting of diagnostic studies over time. When we repeating this search in October 2009, we observed that this number increased to 50%, probably due to the adoption of the *Uniform Requirements for Manuscripts Submitted to Biomedical Journal* (URM) created by the International Committee of Medical Journal Editors (ICMJE), which recommends authors to use “reporting guidelines relevant to their specific research design”, such as STARD.[29] Despite the substantial increase in the proportion of journals recommending the use of STARD, this proportion is still far from ideal.

Decreasing the burden of TB, malaria and HIV is a priority worldwide, and the provision of universal, high-quality and affordable diagnostic tests to affected populations is the first key step to achieve this goal. Regulatory authorities, policy makers and healthcare professionals frequently use diagnostic accuracy studies to decide which test should be implemented in a particular setting. However, choices based on biased study results may lead to detrimental consequences.

Lack of methodological rigour in diagnostic trials is a cause for concern as it may prove to be a major hurdle for effective application of diagnostics in controlling TB, malaria and HIV. Depending on how the presence of bias affects the estimates of diagnostic accuracy, a large number of patients could be harmed by not being properly diagnosed and consequently not receiving adequate care. [16], [17] Furthermore, biased results from poorly designed studies can lead to premature or misguided adoption of tests that may have little or no clinical and public health relevance, and result in incorrect diagnosis and adverse consequences for the patient and/or the healthcare service. A good example of this is widespread use of serological, antibody tests for TB, when all the evidence suggests that they have poor accuracy and have no clinical role.[8] The situation is exacerbated by the fact that most developing countries have poor or nonexistent regulatory mechanisms for marketing and post-marketing surveillance of diagnostics.[30]

Thus, due to the negative implications that biased studies can present, efforts are urgently needed to improve quality of diagnostic research as well as quality of reporting. The more frequent use of tools such as QUADAS and STARD could aid in this process. While not designed with this intent, QUADAS, for example, could be used by researchers as a guideline when designing diagnostic studies, as it describes all the quality elements that should be present in this type of study. QUADAS can also be used as an educational tool, to help train researches in improving research design. STARD can be very useful at the manuscript development stage. However, because voluntary use of tools such as QUADAS and STARD is likely to be limited, their widespread use will probably only happen if more journals explicitly required and mandated authors to use these tools.

While improving diagnostic accuracy studies is a good starting point, efforts must also be made to go beyond test accuracy and generate evidence on patient-important outcomes that can inform policy and guideline development. For example, much of the existing evidence-base in TB is focused on test accuracy [8], [31]. There are limited data on outcomes such as accuracy of diagnostic algorithms (rather than single tests) and their relative contributions to the health care system, incremental value of new tests, impact of new tests on clinical decision-making and therapeutic choices, cost-effectiveness in routine programmatic settings, and impact on patient-important outcomes. Future diagnostic studies must attempt to collect data on these outcomes and not merely focus on test accuracy.

In conclusion, our data suggests that recently published diagnostic studies on commercial tests for TB, malaria and HIV are of moderate to low quality and are poorly reported. Essential methodological and design elements were often either not reported or poorly reported. The more frequent use of tools such as QUADAS and STARD may be necessary to improve methodological quality and reporting of future diagnostic accuracy studies in infectious diseases. This may happen only when more journals require authors to use instruments such as STARD.
